# Trends and Disparities in Firearm Fatalities in the United States, 1990-2021

**DOI:** 10.1001/jamanetworkopen.2022.44221

**Published:** 2022-11-29

**Authors:** Chris A. Rees, Michael C. Monuteaux, Isabella Steidley, Rebekah Mannix, Lois K. Lee, Jefferson T. Barrett, Eric W. Fleegler

**Affiliations:** 1 Division of Pediatric Emergency Medicine, Emory University School of Medicine, Atlanta, Georgia; 2 Division of Emergency Medicine, Children’s Healthcare of Atlanta, Atlanta, Georgia; 3 Division of Emergency Medicine, Boston Children’s Hospital, Boston, Massachusetts; 4 Department of Pediatrics, Harvard Medical School, Boston, Massachusetts; 5 Brown University, Providence, Rhode Island; 6 Division of Pediatric Emergency Medicine, Children’s Hospital at Montefiore, Bronx, New York; 7 Department of Pediatrics, Albert Einstein College of Medicine, New York, New York

## Abstract

**Importance:**

Firearm fatality rates in the United States have reached a 28-year high. Describing the evolution of firearm fatality rates across intents, demographics, and geography over time may highlight high-risk groups and inform interventions for firearm injury prevention.

**Objective:**

To understand variations in rates of firearm fatalities stratified by intent, demographics, and geography in the US.

**Design, Setting, and Participants:**

This cross-sectional study analyzed firearm fatalities in the US from 1990 to 2021 using data from the Centers for Disease Control and Prevention. Heat maps, maximum and mean fatality rate graphs, and choropleth maps of county-level rates were created to examine trends in firearm fatality rates by intent over time by age, sex, race, ethnicity, and urbanicity of individuals who died from firearms. Data were analyzed from December 2018 through September 2022.

**Main Outcomes and Measures:**

Rates of firearm fatalities by age, sex, race, ethnicity, urbanicity, and county of individuals killed stratified by specific intent (suicide or homicide) per 100 000 persons per year.

**Results:**

There were a total of 1 110 421 firearm fatalities from 1990 to 2021 (952 984 among males [85.8%] and 157 165 among females [14.2%]; 286 075 among Black non-Hispanic individuals [25.8%], 115 616 among Hispanic individuals [10.4%], and 672 132 among White non-Hispanic individuals [60.5%]). All-intents total firearm fatality rates per 100 000 persons declined to a low of 10.1 fatalities in 2004, then increased to 14.7 fatalities (45.5% increase) by 2021. From 2014 to 2021, male and female firearm homicide rates per 100 000 persons per year increased from 5.9 to 10.9 fatalities (84.7% increase) and 1.1 to 2.0 fatalities (87.0% increase), respectively. Firearm suicide rates were highest among White non-Hispanic men aged 80 to 84 years (up to 46.8 fatalities/100 000 persons in 2021). By 2021, maximum rates of firearm homicide were up to 22.5 times higher among Black non-Hispanic men (up to 141.8 fatalities/100 000 persons aged 20-24 years) and up to 3.6 times higher among Hispanic men (up to 22.8 fatalities/100 000 persons aged 20-24 years) compared with White non-Hispanic men (up to 6.3 fatalities/100 000 persons aged 30-34 years). Males had higher rates of suicide (14.1 fatalities vs 2.0 fatalities per 100 000 persons in 2021) and homicide (10.9 fatalities vs. 2.0 fatalities per 100 000 persons in 2021) compared with females. Metropolitan areas had higher homicide rates than nonmetropolitan areas (6.6 fatalities vs 4.8 fatalities per 100 000 persons in 2021). Firearm fatalities by county level increased over time, spreading from the West to the South. From 1999 to 2011 until 2014 to 2016, fatalities per 100 000 persons per year decreased from 10.6 to 10.5 fatalities in Western states and increased from 12.8 to 13.9 fatalities in Southern states.

**Conclusions and Relevance:**

This study found marked disparities in firearm fatality rates by demographic group, which increased over the past decade. These findings suggest that public health approaches to reduce firearm violence should consider underlying demographic and geographic trends and differences by intent.

## Key Points

**Question:**

How have firearm fatality rates varied over a 32-year period in the United States?

**Findings:**

In this cross-sectional study of 1 110 421 firearm fatalities, all-intent firearm fatality rates declined to a low in 2004, then increased 45.5% by 2021. Firearm homicides were highest among Black non-Hispanic males, and firearm suicide rates were highest among White non-Hispanic men ages 70 years and older.

**Meaning:**

This study found marked disparities in firearm fatality rates between men and women and by racial and ethnic group, and these disparities increased in recent years.

## Teaser

This cross-sectional study investigates rates of firearm fatalities by intent, age, sex, race, ethnicity, and urbanicity of individuals killed from 1990 to 2021.

## Introduction

Each day, more than 100 firearm deaths occur in the United States.^1^ In 2021, there were 48 953 fatalities from firearms, the highest number of firearm deaths recorded since the Centers for Disease Control and Prevention (CDC) began tracking injury fatalities in 1981.^2^ Previous study findings suggested a recent increase in overall firearm-related mortality rates, and firearms are now the leading cause death in youths aged 1 to 19 years, accounting for 20% of adolescent deaths.^3,4,5,6,7^

In 2019, firearm injuries and fatalities cost an estimated $410 billion in medical costs, work loss, quality of life lost, and total value of life loss.^8^ Prior to the COVID-19 pandemic, an estimated 393 million privately owned firearms were distributed among 40% of US homes.^9,10,11^ Firearms sales surged during the COVID-19 pandemic, with an estimated 7.5 million new firearm owners, and 5.4 million homes previously without firearms now contain firearms.^12^ The increase in firearm ownership was associated with the exposure of more than 16 million people to firearms in the home for the first time.^12^

Despite the large burden of firearm fatalities and the ubiquitous availability of firearms in the US, a contemporary analysis, including age, sex, race, ethnicity, and urbanicity of individuals killed by firearms, is lacking, to our knowledge. Moreover, prior studies have not included trends in firearm fatalities during the COVID-19 pandemic. Such understanding may inform interventions to decrease firearm fatalities by targeting populations in specific geographic areas who have higher rates of firearm fatalities from homicide or suicide. To this end, our objective was to use heat maps, novel maximum and mean fatality rate graphs, and choropleth maps of county-level rates to graphically describe the multidimensional evolution of firearm fatality rates across the US stratified demographically and by intent from 1990 to 2021.

## Methods

We conducted a cross-sectional study using the CDC Web-based Injury Statistics Query and Reporting System (WISQARS) Fatal Injuries Reports, Compressed Mortality File (CMF), and Wide-ranging Online Data for Epidemiologic Research (WONDER) databases to evaluate trends in firearm fatalities.^2,13,14^ The Boston Children’s Hospital Institutional Review Board exempted this study from review because it only analyzed fatality data and determined that participant consent was not needed because all data were obtained from deidentified, publicly available sources. We followed the Strengthening the Reporting of Observational Studies in Epidemiology (STROBE) reporting guideline.

We extracted the national number of firearm deaths and firearm fatality rates per 100 000 persons per year from WISQARS and WONDER in 5-year age groups by sex, race, ethnicity, and metropolitan designation annually from 1990 to 2021. Denominators were the annual number of individuals in each demographic and age group. Race was classified by WISQARS as Black or White using data derived from death certificates. Because the numbers of fatalities by 5-year age group and intent were too low to maintain anonymity, racial categories of American Indian or Alaska Native, Asian or Pacific Islander, and other were excluded. Ethnicity was classified by WISQARS as Hispanic or non-Hispanic. The urbanicity of the location of firearm fatalities (metropolitan vs nonmetropolitan) was classified based on the 2013 National Center for Health Statistics county-based urbanization classifications (available since 1999).^15^ We obtained firearm fatality rates stratified by intent (ie, suicide and homicide) per WISQARS and WONDER.

We extracted county-level firearm fatality rates from the CMF from 1999 to 2016 and population data from the US Census. The CMF stopped providing data at the county level after 2016, and data from WONDER at the county level were too incomplete to extract for 2017 to 2021.^16,17^ Fatality data from WISQARS, WONDER, and CMF were compiled from all available death certificate data collected by state registries and provided to the National Vital Statistics System.

### Statistical Analysis

Because counts were intended to capture all deaths in the US population rather than a sample drawn from a population, inferential statistical tests were not performed. Differences between and within groups were interpreted as population-level outcomes. Heat maps have been used to better understand the multidimensional nature of evolving epidemics, including US trends in the opioid epidemic.^18,19^ We created scaled heat maps of annual firearm fatality rates per 100 000 persons per year stratified by 5-year age groups, sex, race, and ethnicity over the entire 32-year study period and urbanicity over a 23-year period based on available data. We mapped intent of firearm fatalities (ie, all intents, suicide, or homicide) to elucidate variations in trends of firearm fatality rates across demographic groups. To make the data comparable across all groups within a particular intent category, we set the highest rate in each intent at the rate of the age group with the maximum annual rate per 100 000 persons for the total population by intent. We plotted year on the x-axis, age group on the y-axis, and annual sex-, race-, ethnicity-, and urbanicity-specific firearm fatality rates in the body of the heat maps in a graded manner with coloring to indicate rates per 100 000 persons. Figures were created using Microsoft Excel for Mac version 16.43 (Microsoft).

While heat maps capture trends over time and across demographic groups by age, their standardization to the total population results in loss of granular data about the magnitude of differences between race and ethnicity groups. To demonstrate these differences, we created maximum crude and mean crude fatality rate line graphs to examine trends in annual firearm fatalities over time by age group, sex, race, ethnicity, and urbanicity of individuals killed. Graphing the maximum crude rate (ie, the age group with the highest fatality rate in a given year) is a novel approach to understanding the extremes of firearm fatalities and better captures disparities between demographic groups than use of mean crude fatality rates. Mean crude fatality rates across age groups were calculated annually for each demographic group.

We created choropleth maps that provided geospatial visualization of trends in firearm fatalities by intent by race, ethnicity, and sex. We created maps of county-level 3-year fatality rates per 100 000 persons. To examine changes over time within intent type, we determined the fifth and ninety-fifth percentile of intent-specific fatality rates from 1999 to 2001 and divided this range by 10 to create a color-coded scale (with <fifth and >ninety-fifth percentiles as additional categories). We applied this scale to the remaining 3-year graphs within each intent. For all-intents maps with rates by race, ethnicity, and sex, we used a similar procedure using White non-Hispanic males and White non-Hispanic females in 1999 to 2001 as reference groups so comparisons across racial and ethnic groups were possible using baseline data from the beginning of the period to track changes over time. Maps were created using STATA statistical software version 16.0 (StataCorp). Data were analyzed from December 2018 through September 2022.

## Results

**Figure 1.**
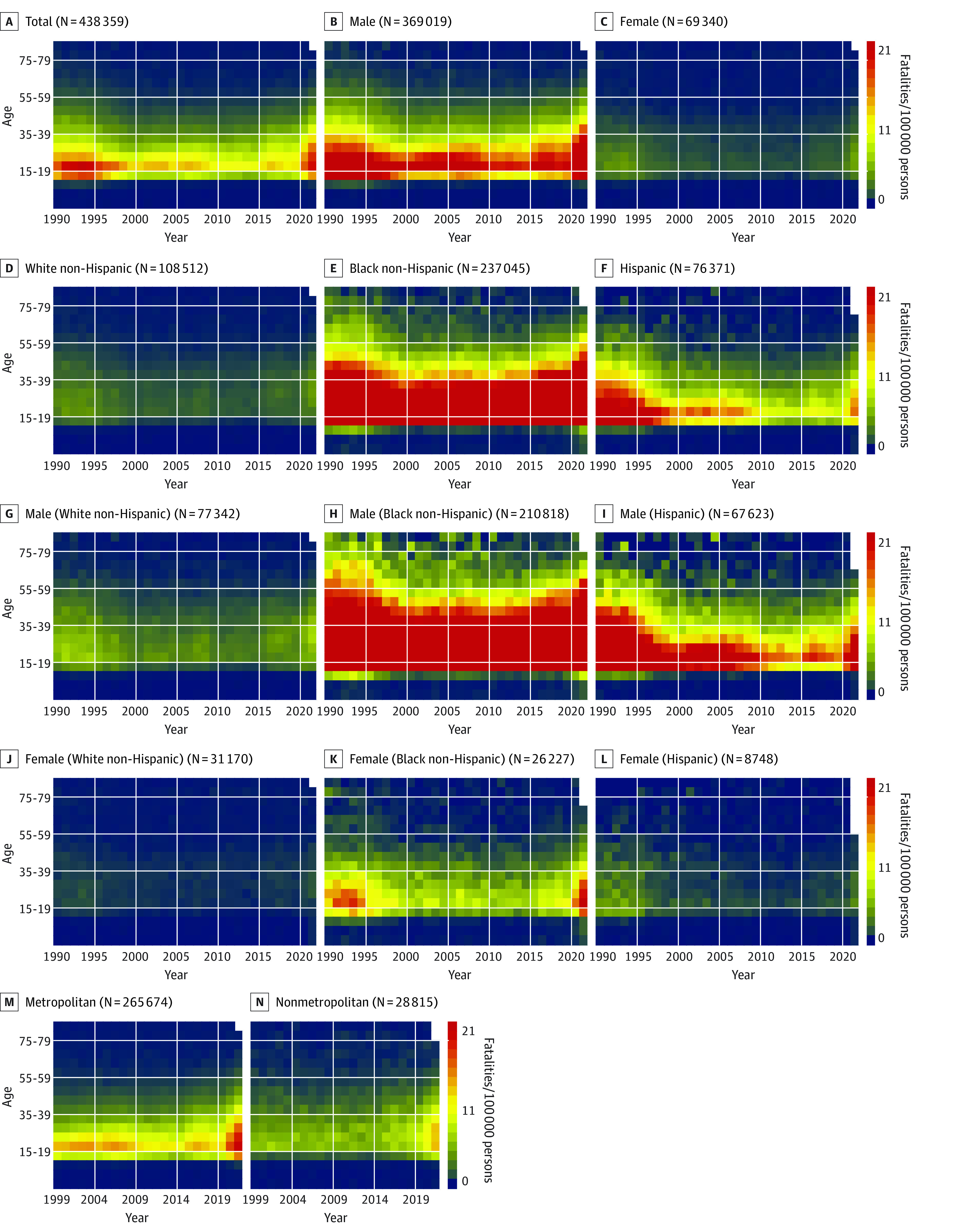
Heat Maps of Age- and Year-Specific Firearm Homicide Fatality Rates Annual firearm homicide fatality rates for the total group and by sex, race, ethnicity, and urbanicity are presented in the body of the heat maps in a graded manner. The coloring scale indicates rates per 100 000 persons per year. Ranges were determined by the minimum and maximum of total homicide fatality rates from panel A. The number of total homicide fatalities is included with the title of each heat map. Urbanicity data were available only from 1999 to 2021. Empty spaces in 2021 indicate that data were not available for those age groups.

**Figure 2.**
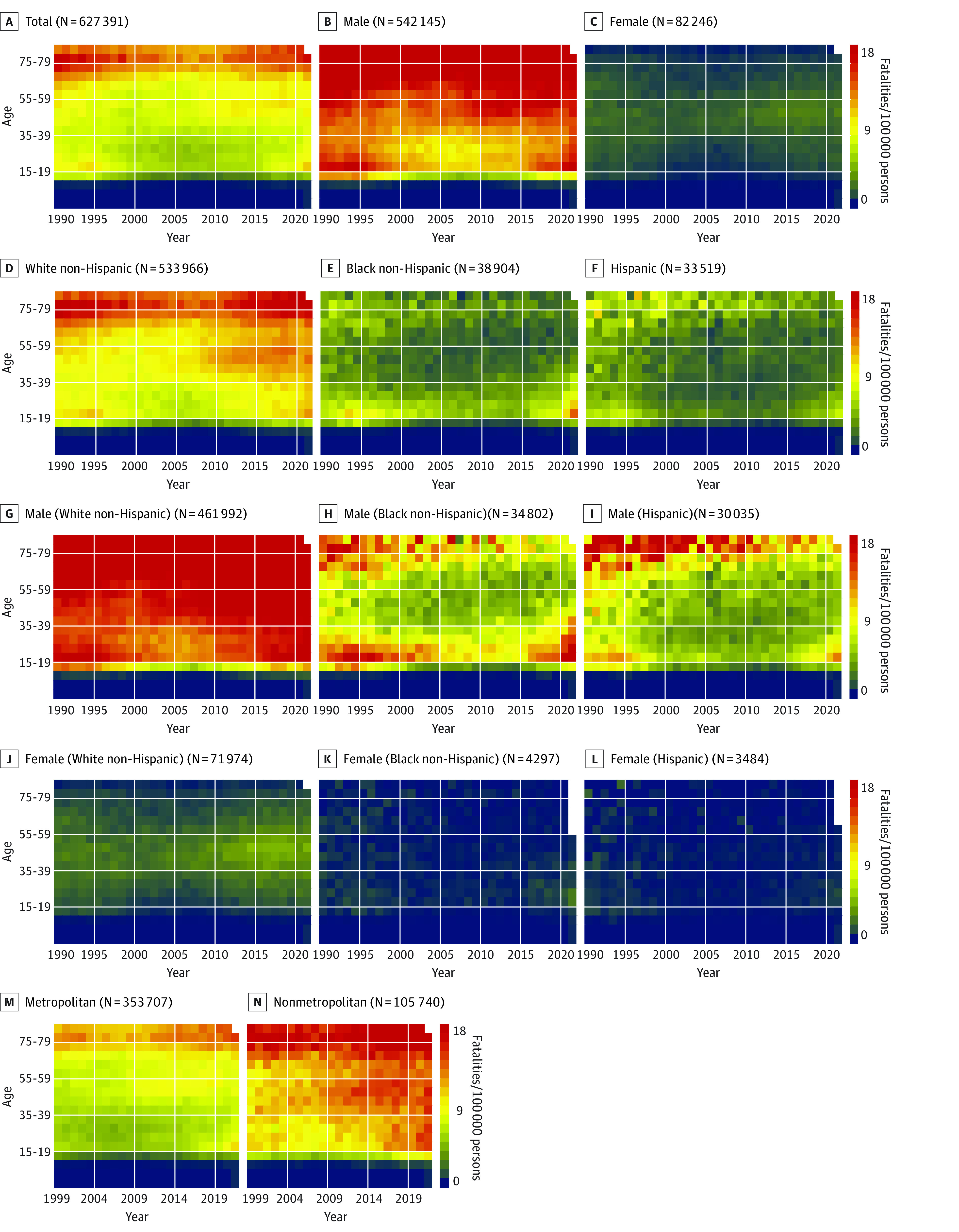
Heat Maps of Age- and Year-Specific Firearm Suicide Fatality Rates Annual firearm suicide fatality rates for the total group and by sex, race, ethnicity, and urbanicity are presented in the body of the heat maps in a graded manner. The coloring scale indicates rates per 100 000 persons per year. Ranges were determined by the minimum and maximum of total suicide fatality rates from panel A. The number of total suicide fatalities is included with the title of each heat map. Urbanicity data were available only from 1999 to 2021. Empty spaces in 2021 indicate that data were not available for those age groups.

There were 1 110 421 firearm fatalities in the US from 1990 to 2021 (952 984 among males [85.8%] and 157 165 among females [14.2%]; 286 075 among Black non-Hispanic individuals [25.8%], 115 616 among Hispanic individuals [10.4%], and 672 132 among White non-Hispanic individuals 60.5%]). Among all demographic groups combined, all-intents annual firearm fatality rates per 100 000 persons per year initially declined from 14.9 fatalities in 1990 to 10.1 fatalities in 2004 (eFigure 1 in Supplement 1). Rates then increased beginning in 2005, reaching a 28-year high in 2021 of 14.7 fatalities per 100 000 persons. Within 5-year age groups, fatalities reached an apogee of 28.7 fatalities per 100 000 persons in 2021 among those aged 20 to 24 years (eFigure 2 in Supplement 2). In 2021, this was associated with peaks in homicides among Black non-Hispanic men (141.8 fatalities/100 000 persons aged 20-24 years) and suicides among White non-Hispanic men (45.2 fatalities/100 000 persons aged 80-84 years) and Black non-Hispanic men (24.5 fatalities/100 000 persons aged 20-24 years) (Figure 1 and Figure 2). Homicide and suicide rates per 100 000 persons per year among Hispanic males continued to decrease over 30 years but then increased in 2019 to 2021.

### Firearm Fatality Rate Heat Maps

Heat maps of all-intents firearm fatality rates showed declines in firearm fatality rates per 100 000 persons per year among those aged 20 to 24 years, from 17.8 fatalities in 1990 to 1995 to a low of 12.3 fatalities in 2006, with a noted increase to 16.8 fatalities in 2021 (eFigure 1 in Supplement 1). However, when visualized by urbanization status, annual firearm suicide rates remained high and increased over time in nonmetropolitan areas among individuals aged 35 years or older (Figure 2). In contrast, metropolitan areas had consistently high rates of homicide over time, especially among individuals aged 20 to 29 years (Figure 1). Metropolitan areas had higher homicide rates than rural areas (6.6 fatalities vs 4.8 fatalities per 100 000 persons in 2021). Comparing all-intents firearm fatality rates over time by sex, males had persistently higher overall annual firearm fatality rates than females, particularly among those aged 20 to 39 years and 70 years or older. Males had higher rates of suicide (14.1 fatalities vs 2.0 fatalities per 100 000 persons in 2021) and homicide (10.9 fatalities vs 2.0 fatalities per 100 000 persons in 2021) compared with females. All-intents annual firearm fatality rates were highest among Black non-Hispanic individuals aged 15 to 39 years; the highest rate was 191.2 fatalities per 100 000 persons in 1991, which declined by half to 96.4 fatalities per 100 000 persons per year, then rebounded to 169.8 fatalities per 100 000 persons among Black non-Hispanic individuals aged 20 to 24 years in 2021.

White non-Hispanic men had the highest annual all-intents firearm fatality rates in individuals aged 75 years and older, at 39.6 fatalities per 100 000 persons in 2021 (eFigure 1 in Supplement 1). Black non-Hispanic men aged 15 to 44 years had the highest annual all-intents firearm fatality rates (126.7 fatalities/100 000 persons in 2021), which was mostly attributable to homicides (107.9 fatalities/100 000 persons in 2021). Annual firearm fatality rates from suicide were highest among White non-Hispanic men aged 80-84 years (52.3 fatalities per 100 000 persons in 1990), which decreased to 33.2 fatalities per 100 000 persons in 2007 (36.5% decrease) and rebounded to 46.8 fatalities per 100 000 persons in 2021 (41.0% increase) (Figure 1). Rates of homicide were highest among Black non-Hispanic men aged 20 to 24 years, at 182.7 fatalities per 100 000 persons in 1993, which decreased to 81.4 fatalities per 100 000 persons in 2014 (55.4% decrease) and rebounded to 141.8 fatalities per 100 000 persons in 2021 (74.2% increase) (Figure 2). By 2021, maximum rates of firearm homicide were up to 22.5 times higher among Black non-Hispanic men (up to 141.8 fatalities/100 000 persons aged 20-24 years) and up to 3.6 times higher among Hispanic men (up to 22.8 fatalities/100 000 persons aged 20-24 years) compared with White non-Hispanic men (up to 6.3 fatalities/100 000 persons aged 30-34 years). Hispanic males had peak rates of annual firearm fatalities per 100 000 persons in 1992 (71.7 fatalities), which declined to 21.3 fatalities in 2014 (70.3% decrease) but rebounded to 37.5 fatalities in 2021 (76.1% increase).

White non-Hispanic females had increased annual firearm fatality rates over time, associated with increases in suicide, but overall significantly lower rates than males. Black non-Hispanic females had increased rates of homicide per 100 000 persons per year in the 1990s, which decreased from 18.7 fatalities in 1994 to 6.2 fatalities in 2010, then more than tripled to 22.0 fatalities in 2021.

### Maximum and Mean Firearm Fatality Rates

The mean total firearm fatality rate per 100 000 persons per year from all intents peaked at 15.2 fatalities in 1991, declined to 10.1 fatalities in 2004, then increased to 14.7 fatalities in 2021 (45.5% increase) (eFigure 2 in Supplement 1). The maximum rate from total all-intents firearm fatalities during that period was 33.8 fatalities per 100 000 persons. Maximum fatality rates per 100 000 persons, the measure of disparities at the extreme ends of fatality rates, showed striking variation between males and females. In 2021, the difference between males and females was 28.4 fatalities vs 4.5 fatalities per 100 000 persons (6.3-fold difference) for homicides and 37.8 fatalities vs. 3.5 fatalities per 100 000 persons (10.8-fold difference) for suicides.

**Figure 3.**
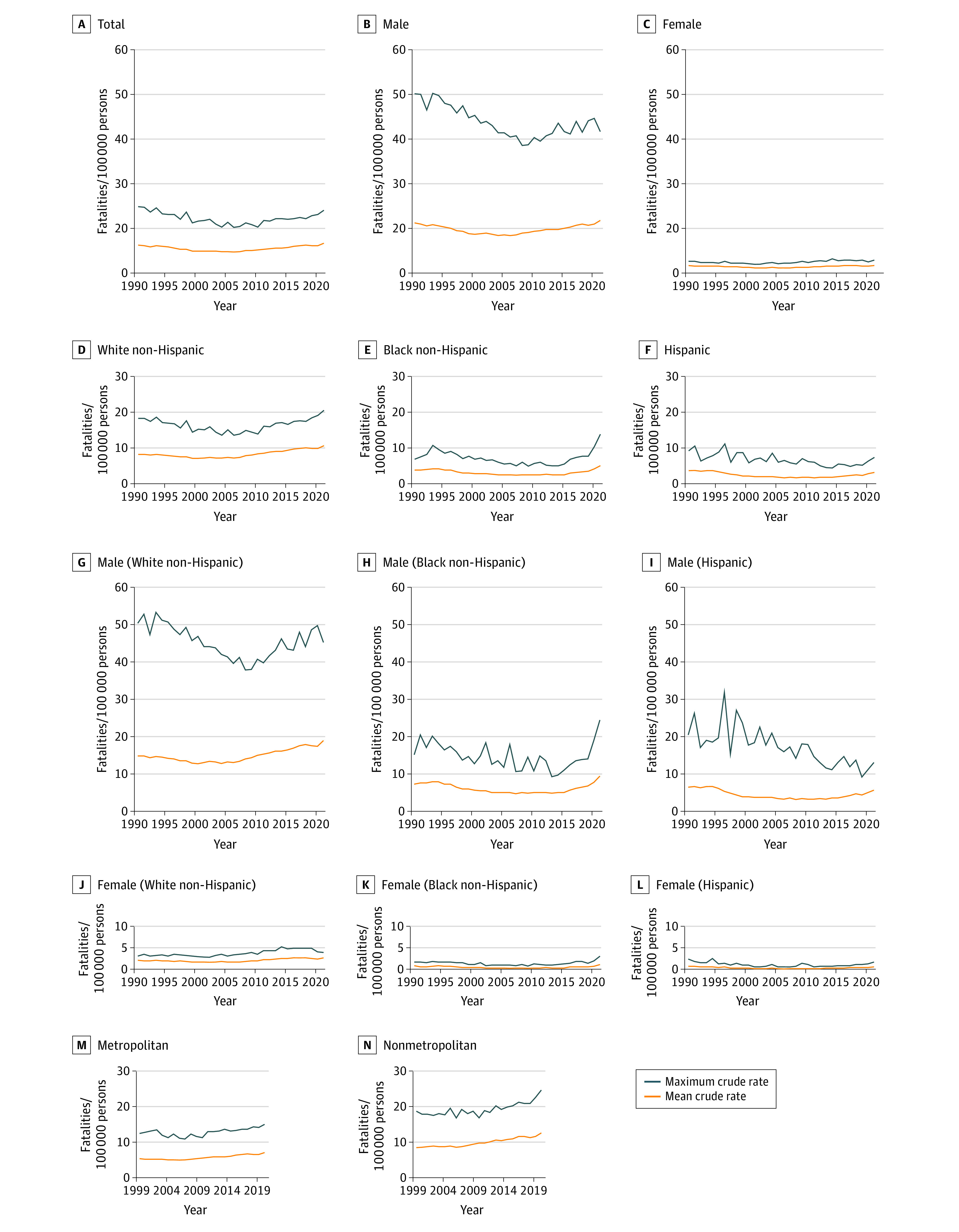
Maximum and Mean Firearm Suicide Fatality Rates Maximum fatality rates are the highest rate for any age group reported annually within a specific demographic stratum. Mean fatality rates are mean rates over the study period calculated each year. The upper limit of the y-axis was set as the highest value of the maximum rate. Urbanicity data were available only from 1999 to 2021.

Since 2010, all-intents firearm fatality rates per 100 000 persons increased among females (from 4.2 fatalities in 2010 to 7.2 fatalities in 2021, a 71.4% increase), associated with higher annual suicide rates (eFigure 2 in Supplement 1; Figure 3). White non-Hispanic populations had a decrease in all-intents firearm fatality mean rates per 100 000 persons beginning in the early 2000s (from 11.6 fatalities in 1991 to 9.1 fatalities in 2000), but since 2010, annual mean firearm fatality rates have increased, peaking at 13.1 fatalities in 2021. Black non-Hispanic populations had an overall decrease in annual mean firearm fatality rates per 100 000 persons per year by all intents (from 36.8 fatalities in 1993 to 19.8 fatalities in 2000), although starting in 2013, annual all-intents, homicide, and suicide rates of firearm fatalities per 100 000 persons per year increased, with peaks in 2021 (36.7 fatalities, 30.8 fatalities, and 5.2 fatalities, respectively).

**Figure 4.**
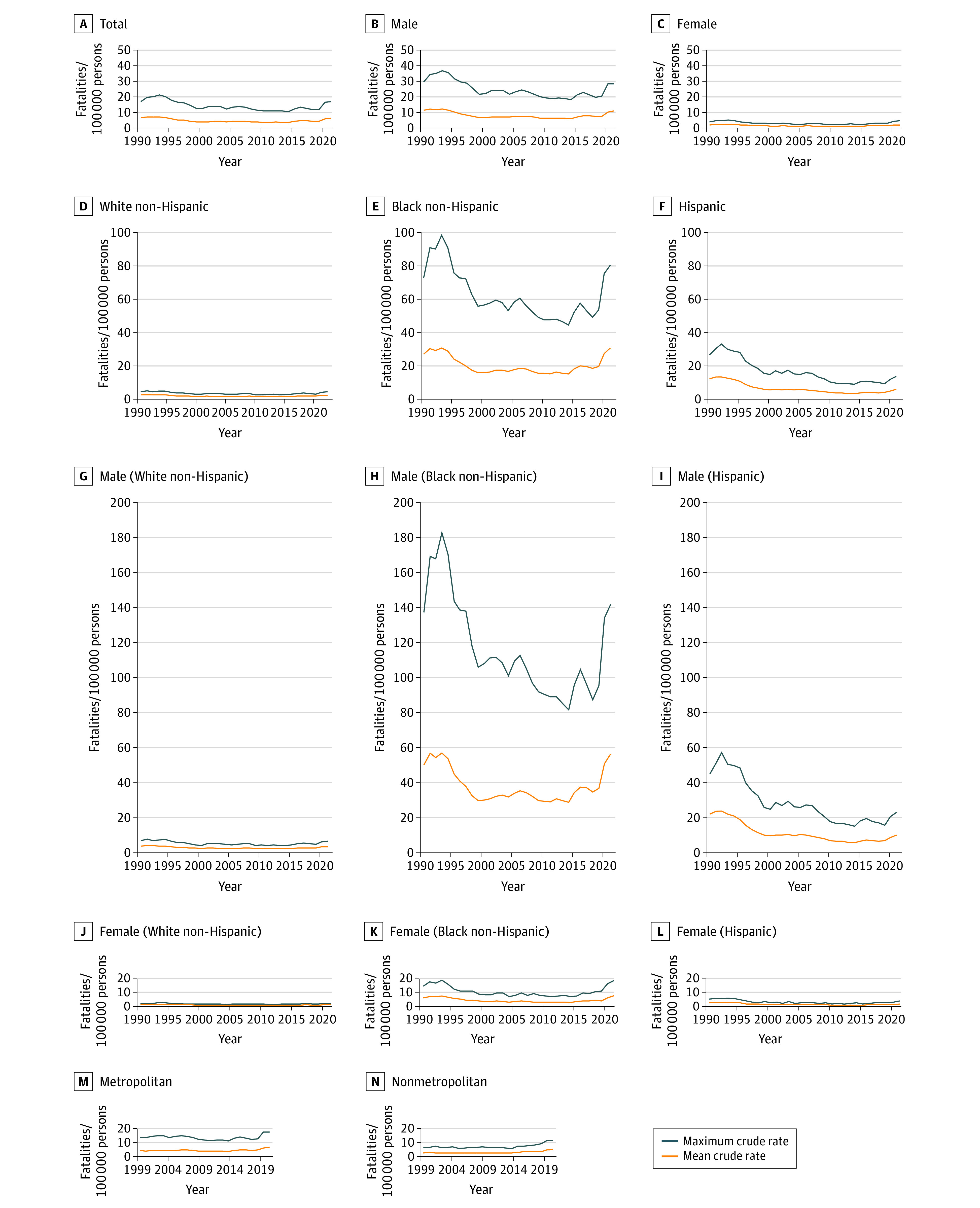
Maximum and Mean Firearm Homicide Fatality Rates Maximum fatality rates are the highest rate for any age group reported annually within a specific demographic stratum. Mean fatality rates are mean rates over the study period calculated each year. The upper limit of the y-axis was set as the highest value of the maximum rate. Urbanicity data were available only from 1999 to 2021.

Disparities were apparent in firearm fatality rates by sex, race, and ethnicity. From 2014 to 2021, male and female firearm homicide mean rates per 100 000 increased from 5.9 to 10.9 fatalities (84.7% increase) and 1.1 to 2.0 fatalities (87.0% increase), respectively. In 2021, White non-Hispanic males had the highest maximum firearm fatality rates per 100 000 persons from suicide (45.2 fatalities) compared with Black non-Hispanic males (24.5 fatalities) and Hispanic males (13.2 fatalities) (Figure 3). In contrast, Black non-Hispanic males had higher mean and maximum fatality rates per 100 000 persons for homicide (mean, 56.2 fatalities; maximum, 141.8 fatalities in 2021) compared with White non-Hispanic (mean, 3.0 fatalities; maximum, 6.3 fatalities in 2021) and Hispanic (mean, 9.7 fatalities; maximum, 22.8 fatalities in 2021) males (Figure 4). The rates were also higher for all-intents fatalities (eFigure 2 in the Supplement).

There was a more than 2-fold increase in suicide-related annual firearm fatality rates per 100 000 persons per year among Black non-Hispanic females after 2015 (from 1.4 fatalities in 2015 to 3.2 fatalities in 2021). From 1990 to 2007, mean rates per 100 000 persons per year in this group decreased, then increased to 2021 rates. All-intents fatalities decreased from 3.5 to 1.5 fatalities, then increased to 2.4 fatalities. Suicide fatalities decreased from 0.9 to 0.3 fatalities, then increased to 0.8 fatalities. Homicide fatalities decreased from 2.6 to 1.1 fatalities, then increased to 1.5 homicide fatalities. Disparities in maximum fatality rates per 100 000 persons per year among females were highest by homicide, with Black non-Hispanic females dying in 2021 at a maximum rate of 18.2 fatalities, Hispanic females at 3.7 fatalities, and White non-Hispanic females at 2.2 fatalities (Figure 4).

### Geographic Distribution of Firearm Fatality Rates

**Figure 5.**
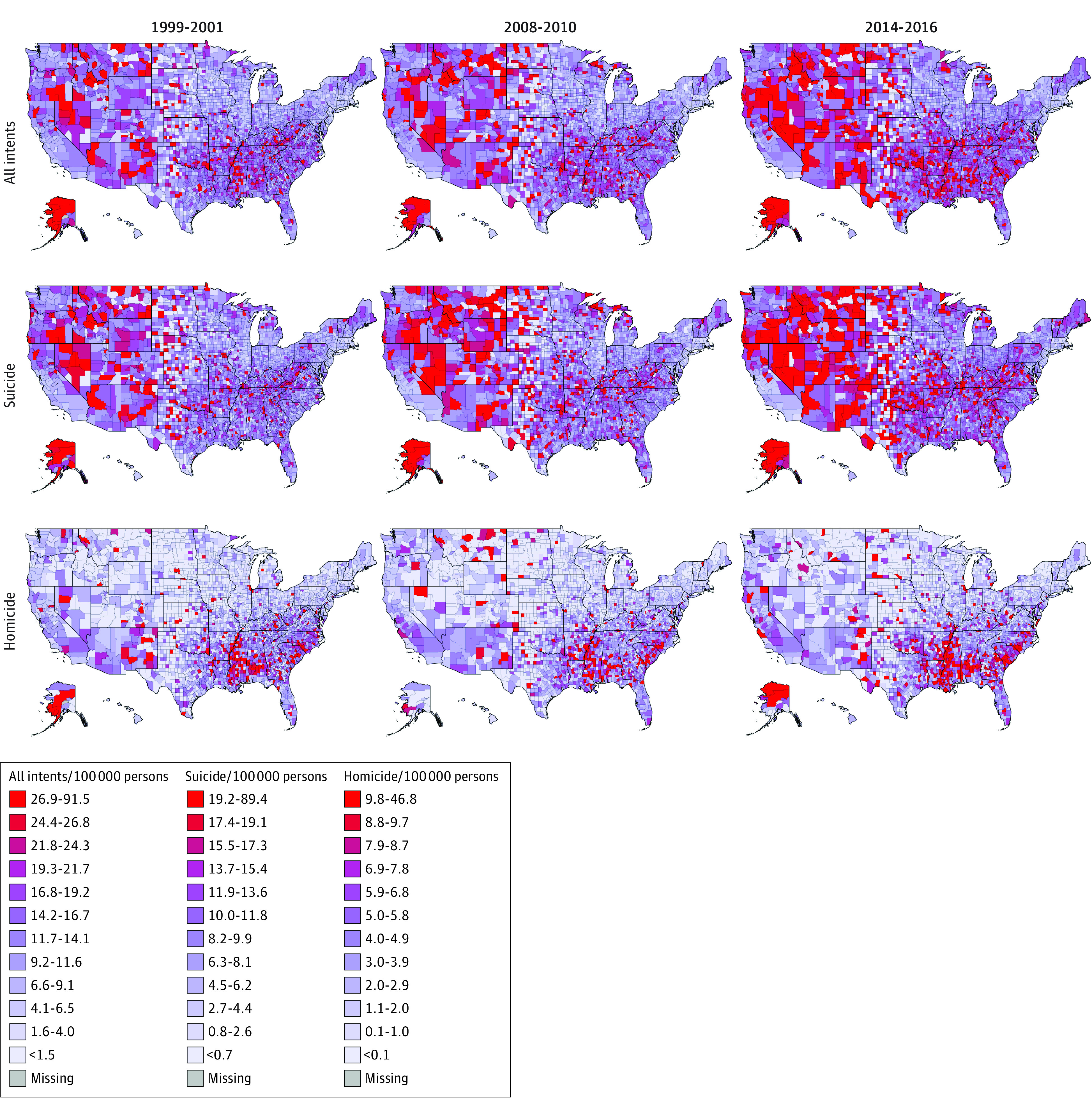
Geographic Distribution of Firearm Fatalities at County Level Comparable county-level data were not available for 2017 to 2021.

Choropleth maps of county-level firearm fatality rates by all intents demonstrated higher rates beginning in Western states that increased toward the South over time (Figure 5). From 1999 to 2011 until 2014 to 2016, fatalities per 100 000 persons decreased from 10.6 to 10.5 fatalities in Western states and increased from 12.8 to 13.9 fatalities in Southern states. Firearm suicide fatality rates had a similar pattern. In contrast, firearm homicide fatality rates were concentrated in Southern states and Alaska and increased over time.

## Discussion

In this multidimensional, nationally representative cross-sectional study of annual US firearm fatalities over a 32-year period, there were notable disparities in firearm fatalities by sex, race, ethnicity, and geography. Using heat maps, maximum and mean fatality line graphs, and choropleth maps of county-level firearm fatality rates, we were able to visualize this evolution of firearm fatalities over time, its variability by age, and disparities by sex, race, ethnicity, and region. Although all-intents firearm fatality rates initially decreased, we found a recent increase in each intent category. Moreover, stratified analyses demonstrated differential patterns in at-risk subpopulations. Annual firearm homicide fatality rates among Black non-Hispanic and Hispanic men aged 20 to 40 years were substantially higher than other demographic groups. Annual firearm suicide fatality rates were highest among White non-Hispanic men aged 70 years or older and increased over time in females. The choropleth maps of county-level rates demonstrate that all-intents firearm fatalities increased over the study period and increased across the country from the West to the South, whereas homicides remained concentrated in the South.

Our heat maps allow for a novel, multidimensional visualization to provide granular detail of the evolving epidemic of firearm violence in the US across a 32-year period. This type of data representation also facilitates the conceptualization of firearm fatality rates varying by age group, sex, race, ethnicity, and intent in 1 picture over time. Heat maps of the US have been used to visualize the evolution of opioid overdoses^18,19^ and variations in hypertension, diabetes, and smoking by geographic region, race, and ethnicity.^20^ Such an approach has not been used to evaluate the growing firearm epidemic in the US, to our knowledge. We also created maximum and mean fatality rate line graphs given that graphing maximum fatality rates allows one to visualize the severity of the problem in the extremes. In graphing maximum and mean annual firearm fatality rates by sex, race, and ethnicity in the US, we were able to further identify demographic groups that may be in need of targeted interventions for firearm injury prevention.

Although prior studies found that firearm fatality rates among all demographic groups remained fairly constant from 2000 to 2010,^21,22^ our study showed a 45.5% increase in all-intents firearm fatalities from 2004 to 2021. This aligns with data from the CDC WONDER database reporting a 28% increase in firearm deaths among children and adolescents from 2013 to 2016.^23^ The increase in firearm fatalities has not been equally distributed geographically in the US. Prior study findings suggest that firearm-related suicides may be more common in more impoverished counties.^24,25,26,27^ The maps in our study may serve to demonstrate where interventions may be focused at the state and county level.

There were evident differences in firearm fatalities by race and ethnicity from 1990 to 2021. Black non-Hispanic and Hispanic men aged 20 to 40 years had higher rates of firearm homicides than White non-Hispanic men of the same age. This finding expands on previously reported data in which firearm fatalities, particularly homicides, were more common among young and middle-age Black and Hispanic men compared with White men of the same age.^28,29,30,31,32,33,34^ There is increasing evidence suggesting the association of structural racism, individual and community poverty, and the environment with disparities in health outcomes in the US,^35,36,37,38^ which may provide a partial explanation of our findings. The marked recent increase in firearm fatalities among Black non-Hispanic men aged 20 to 24 years is noteworthy and suggests that intervention is required to reduce this concerning recent trend.

In contrast to Black non-Hispanic men, White non-Hispanic men aged 70 years and older had the highest rates of firearm fatalities from suicide. This finding aligns with a study by Conner et al,^39^ which found that individuals aged 65 years or older were less likely to attempt suicide than those younger than age 25 years but were more likely to die by suicide. That study did not examine trends by race or ethnicity. These findings suggest that suicide prevention efforts in the US may be most beneficial if they target older men. Our finding of higher rates of firearm-related suicide among males compared with females was consistent with prior studies finding that females were more likely to attempt suicide with poisoning, while males more commonly used a more lethal approach to suicide: firearms.^40^ Nonetheless, increasing firearm suicide rates in females over time are concerning. Identifying individuals at risk for suicide who have access to firearms and facilitating lethal means restriction for people with depression or prior suicide attempts may reduce rates of firearm-related suicide.^41,42^

Combined visualization modalities of demographic trends underscore demographic and geographic areas in need of targeted interventions. State-level legislation is 1 such approach. For example, states with more stringent child access–prevention laws,^43^ more comprehensive background checks, and more firearm purchase regulations had fewer firearm-related fatalities.^44,45^ Choropleth maps of county-level rates also suggested hot spots at the county level that may benefit from community-based interventions, such as violence intervention programs, firearm buybacks, safe firearm storage, and suicide prevention programs. State officials may use this information to understand potential interventions to be implemented at the local level.

### Limitations

This study has several limitations. Although overall rates of firearm fatalities were high for American Indian and Alaska Native individuals and low for Asian individuals,^46^ the numbers were too small to report for each group when subdivided by age groups and intent over time and therefore were not addressed in this study. There is the potential for misclassification of intent for firearm fatalities^47^ or changes in how firearm fatalities were coded over time. The WISQARS database does not contain information on owners of firearms or types of firearms used in firearm fatalities. Such information would further allow for targeted interventions designed to reduce the large number of firearm fatalities in the US. We were not able to control for multifactorial secular trends, such as increasing mass school shootings,^48^ increased firearm ownership, the COVID-19 pandemic, or changes in storage patterns of firearms over time. Additionally, death certificates used in WISQARS may be incomplete, which may have led to underestimates of firearm fatality rates.

## Conclusions

Using a multidimensional approach over 3 decades in the US, this cross-sectional study found marked disparities in rates of firearm fatalities by sex, race, ethnicity, and region. Homicides were most common among Black non-Hispanic men aged 20 to 40 years and suicides among White non-Hispanic men aged 70 years or older. Annual firearm suicide rates among females increased since 2010. Heat maps and maximum fatality line graphs may provide a robust perspective for visualizing the evolving firearm epidemic in the US. The geographical evolution of firearm fatalities in the US demonstrated concentrated and increased rates moving from the West to the South over time. Our findings suggest that public health approaches to prevent firearm violence must consider underlying demographic trends and differences by intent to reduce disparities and fatalities.

## Notes

Supplement. **eFigure 1.** Age- and Year-Specific All-Intents Firearm Fatality Rates Per 100 000 Persons, 1990-2021 **eFigure 2.** Maximum and Mean Firearm All-Intents Fatality Rates, 1990-2021

Supplement 2.
Data Sharing Statement
