# Supplementary material for: Trends and Disparities in Firearm Fatalities in the United States, 1990-2021
Source: JAMA Netw Open. 2022 Nov 29;5(11):e2244221. doi: 10.1001/jamanetworkopen.2022.44221 (PMC9709653; doi:10.1001/jamanetworkopen.2022.44221)
Supplement: Supplement. — eFigure 1. Age- and Year-Specific All-Intents Firearm Fatality Rates Per 100 000 Persons, 1990-2021 eFigure 2. Maximum and Mean Firearm All-Intents Fatality Rates, 1990-2021 [file jamanetwopen-e2244221-s001.pdf]

Supplemental Online Content

Rees CA, Monuteaux MC, Steidley I, et al. Trends and disparities in firearm fatalities in the United States, 1990-2021. *JAMA Netw Open.* 2022;5(11):e2244221. doi:10.1001/jamanetworkopen.2022.44221

eFigure 1. Age- and Year-Specific All-Intents Firearm Fatality Rates Per 100 000 Persons, 1990-2021
eFigure 2. Maximum and Mean Firearm All-Intents Fatality Rates, 1990-2021

This supplemental material has been provided by the authors to give readers additional information about their work.

© 2022 Rees CA et al. *JAMA Network Open.*

eFigure 1. Age- and Year-Specific All-Intents Firearm Fatality Rates Per 100 000 Persons, 1990-2021*

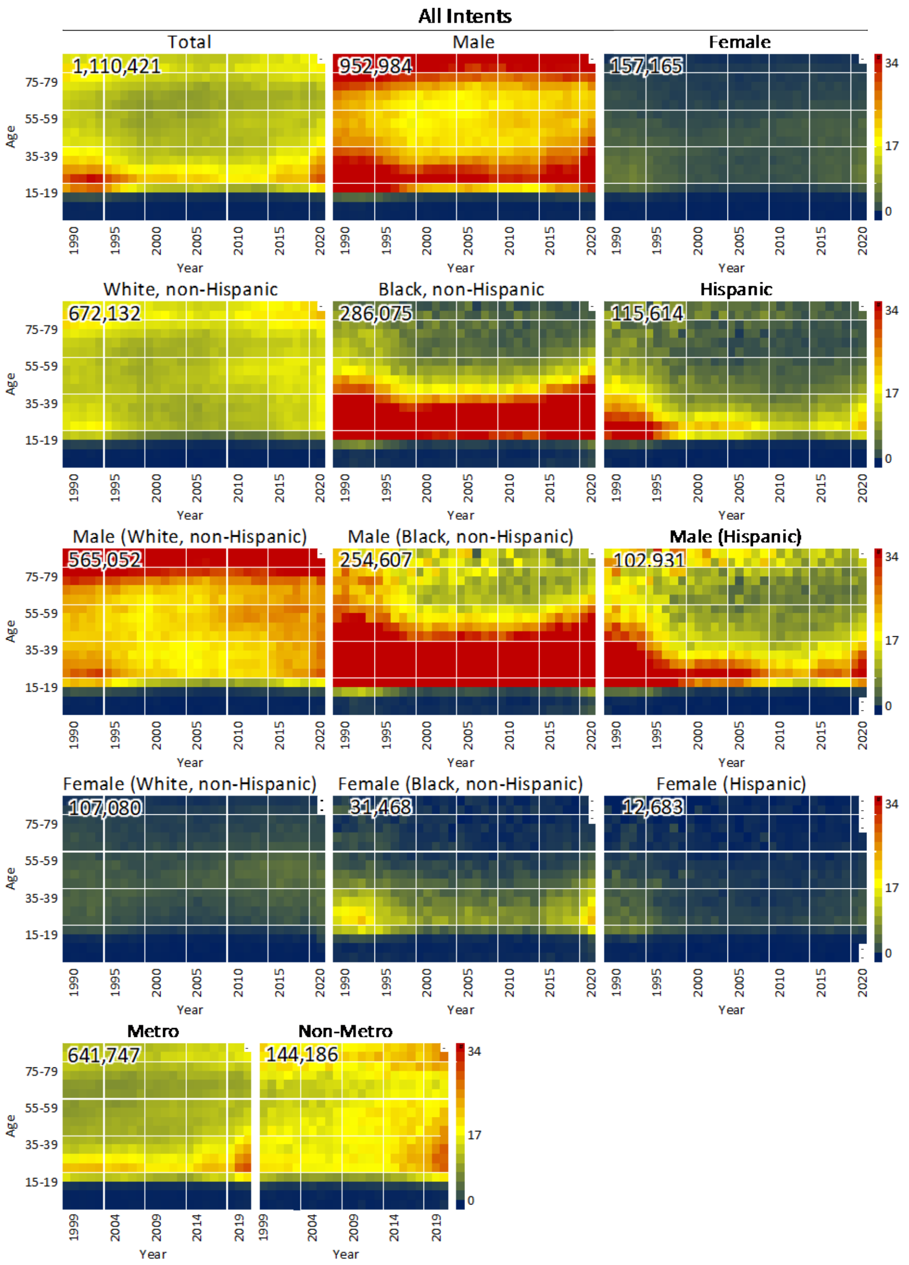

*Year is plotted on the x-axis, age group on the y-axis, and annual firearm all intents fatality rates by total, sex, race, ethnicity and urbanicity in the body of the heatmaps in a graded manner. Coloring scale denotes different rates per 100,000 persons. Ranges determined by minimum and maximum of the total all intents fatality rates. The number of total all intents fatalities is included in the top left corner of each heatmap. Urbanicity data were only available from 1999-2021.

eFigure 2. Maximum and Mean Firearm All-Intents Fatality Rates, 1990-2021*

© 2022 Rees CA et al. *JAMA Network Open.*

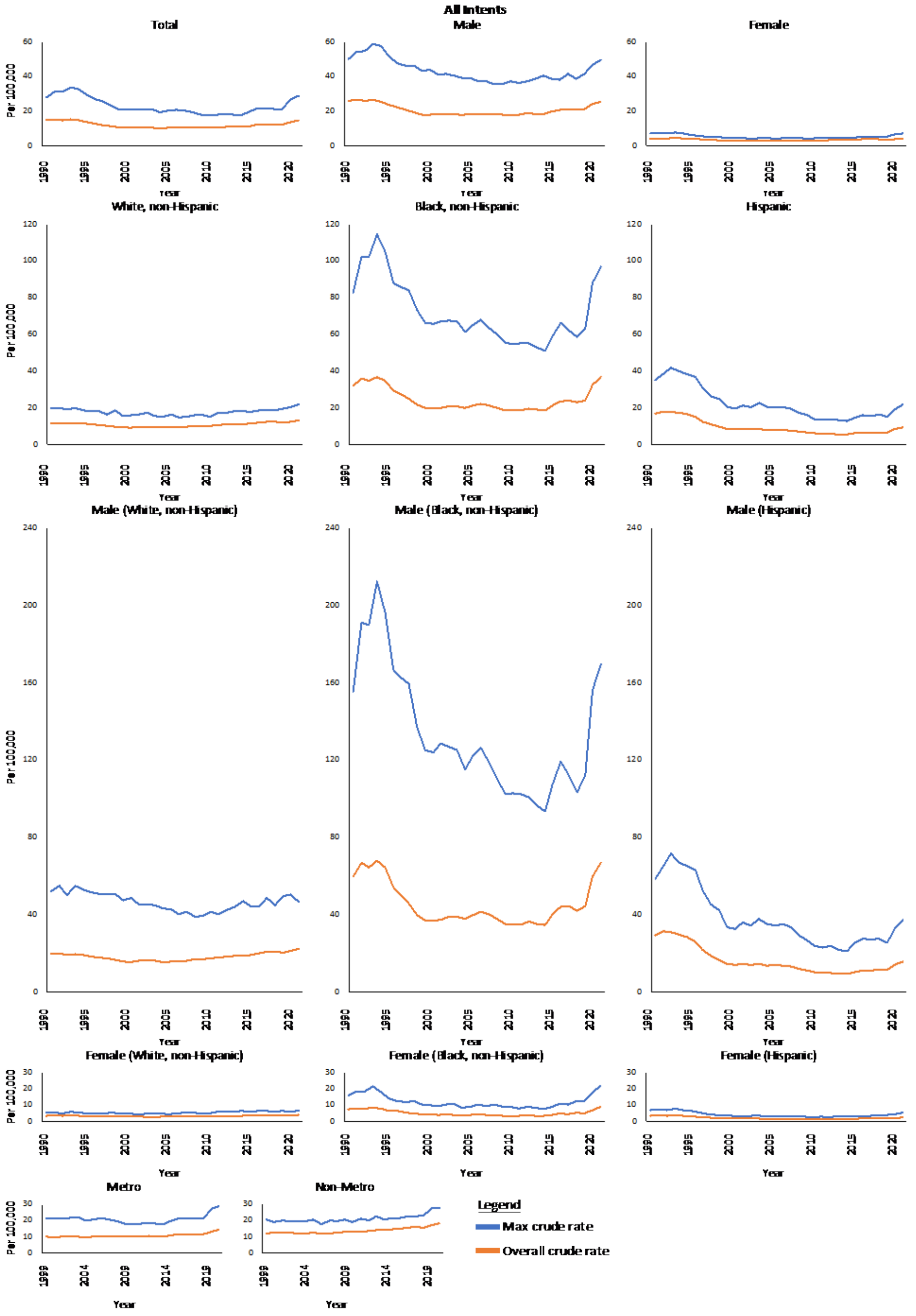

*Maximum fatality rates are the highest rate for any age group reported annually within a specific demographic stratum. Average fatality rates are average rates over the study period calculated each year. The upper limit of the y-axis was set as the highest value of the maximum rate. Urbanicity data were only available from 1999-2021.

© 2022 Rees CA et al. *JAMA Network Open.*
