# Supplementary material for: Trends and Disparities in Firearm Fatalities in the United States, 1990-2021
Source: JAMA Netw Open. 2022 Nov 29;5(11):e2244221. doi: 10.1001/jamanetworkopen.2022.44221 (PMC9709653; doi:10.1001/jamanetworkopen.2022.44221)
Supplement: Supplement 2. — Data Sharing Statement [file jamanetwopen-e2244221-s002.pdf]

Data Sharing Statement

Rees CA, Monuteaux MC, Steidley I, et al. Trends and disparities in firearm fatalities in the United States, 1990-2021. *JAMA Netw Open*. 2022;5(11):e2244221. doi:10.1001/jamanetworkopen.2022.44221

Data
Data available: Yes
Data types: Other (please specify)
Additional Information: All data are publicly available.
How to access data: The dataset used in this study may be made available upon reasonable request to the corresponding author.
When available: With publication

Supporting Documents
Document types: None

Additional Information
Who can access the data: The dataset used in this study may be made available upon reasonable request to the corresponding author.
Types of analyses: For any purpose.
Mechanisms of data availability: The dataset used in this study may be made available upon reasonable request to the corresponding author.

© 2022 Rees CA et al. *JAMA Network Open*.
